# Integrating meteorological and breeding data to predict maize yields using machine learning algorithms

**DOI:** 10.3389/fpls.2025.1722068

**Published:** 2025-11-21

**Authors:** Shaoqiang Wang, Guangcai Wang, Yuchen Wang, Yanbing Wang, Huiguang Wang, Weixian Li, Jianwei Wei, Xuwen Jiang

**Affiliations:** 1 School of Information and Control Engineering, Qingdao University of Technology, Qingdao, China; 2 Hebei Provincial Key Laboratory of Crops Drought Resistance Research, Dryland Farming Institute, Hebei Academy of Agriculture and Forestry Sciences, Hengshui, China; 3 School of Science, Qingdao University of Technology, Qingdao, China; 4 Institute of Cereal and Oil Crops of Hebei Academy of Agriculture and Forestry Sciences, Shijiazhuang, China; 5 School of Electronic and Information Engineering, Langfang Normal University, Langfang, China; 6 Maize Research Institute, Qingdao Agricultural University, Qingdao, China

**Keywords:** meteorological data, breeding value, maize hybrids, machine learning model, artificial intelligence

## Abstract

**Introduction:**

Accurate crop yield prediction is vital for ensuring global food security, particularly amid growing environmental challenges such as climate change. Although deep learning (DL) methods have shown potential in yield prediction, they often demand large datasets and considerable computational resources.

**Methods:**

To address these limitations, this study developed a machine learning (ML) model to predict maize hybrid yields by integrating meteorological data with breeder-level genetic information, specifically breeding values estimated using the best linear unbiased prediction (BLUP) method. Four commonly used ML algorithms—Random Forest (RF), XGBoost, Support Vector Regression (SVR), and Gaussian Process Regression (GPR)—were evaluated and optimized through hyperparameter tuning.

**Results:**

Among these models, the RF algorithm achieved the best performance, with a coefficient of determination (R²) of 0.64, a root mean square error (RMSE) of 1010.59 kg/ha, a mean absolute error (MAE) of 743.89 kg/ha, a relative RMSE (RRMSE) of 10.32%, and a mean absolute percentage error (MAPE) of 8.3%.

**Discussion:**

These results demonstrate that the proposed RF-based model can provide accurate yield predictions for specific maize cultivars under diverse planting conditions. This predictive framework offers practical support for farmers in selecting well-adapted hybrids and serves as a cost-effective, efficient tool for breeders to identify high-yielding maize hybrids optimized for particular environments. Consequently, the model promotes smarter breeding strategies and more precise cultivation recommendations.

## 1 Introduction

Food security is one of the most urgent global challenges today, compounded by environmental degradation, water scarcity, and the increasing threats posed by climate change (Sharma et al., 2022). As the world’s population grows, the demand for agricultural production rises. However, crop yields are highly sensitive to fluctuations in climatic factors such as temperature, precipitation, soil quality, and atmospheric humidity (Arora, 2019; Ortiz-Bobea et al., 2021). These factors, which are subject to environmental changes, complicate efforts to achieve stable crop yields and threaten food supply stability in many regions (Razzaq et al., 2021; Munaweera et al., 2022). As such, the ability to accurately predict crop yield is increasingly crucial for modern breeding programs focused on developing high-yielding and climate-resilient crop varieties (Hafeez et al., 2023).

Despite the growing body of research on crop yield prediction, challenges remain in integrating diverse data sources. Most studies have either focused exclusively on genomic data or meteorological data, often without combining both to enhance predictive accuracy. In particular, limited research has explored integrating breeding values estimated through the best linear unbiased prediction (BLUP) method with time-series meteorological data to predict hybrid-specific yields. This gap in the literature presents an opportunity for this study, which aims to combine these two data types to develop a more accurate, interpretable, and adaptable yield prediction model.

In recent years, the field of crop breeding has shifted from Breeding 3.0 to Breeding 4.0, driven by the integration of advanced technologies such as artificial intelligence (AI) and big data analytics (Shen et al., 2022). Among AI applications in agriculture, machine learning (ML) techniques have shown significant potential in improving crop yield prediction models. Machine learning algorithms, such as decision trees (Papageorgiou et al., 2011), support vector machines (Sujay and Deka, 2014), and K-nearest neighbors (Medar and Rajpurohit, 2014), have successfully enhanced yield forecasting by incorporating both genotypic and environmental data. More sophisticated approaches, including convolutional neural networks (CNNs) and recurrent neural networks (RNNs) (Sun et al., 2019), deep neural networks (DNNs) (Khaki and Wang, 2019), and long short-term memory networks (LSTMs) (Muruganantham et al., 2022), have further improved predictive accuracy. Similar approaches have been used in other crops like wheat, rice, and barley. For example, wheat yield predictions have integrated genomic data with climate variables to improve accuracy (Juliana et al., 2020), and similar approaches for rice have combined weather data with genomic data to forecast yields more reliably (Kumar et al., 2021). These studies highlight the value of integrating breeding and environmental data to enhance yield prediction models. However, a major challenge in adopting machine learning techniques is the scarcity of large, high-quality datasets required to train robust models. This issue is particularly prominent in small- and medium-sized seed companies, which often have limited trial sites and insufficient data for machine learning model development (Tanwar et al., 2019; Sharma and Shivandu, 2024). Collaborative breeding initiatives that pool data from multiple institutions have been proposed to address this problem, but concerns over intellectual property and data privacy pose significant barriers to effective collaboration (Newton et al., 2020). These challenges highlight the need for innovative solutions that enable collaboration without compromising sensitive data (Bachmann et al., 2022).

Traditional crop growth models simulate plant development under specific conditions but often fail to differentiate between genotypes within the same crop species. Deep learning models like CNNs and LSTMs offer high predictive power, but they require large datasets and extensive computational resources. In contrast, the machine learning algorithms used in this study are better suited for moderate-sized datasets, offering improved interpretability—a crucial feature for practical use in plant breeding (Niedbała, 2019). This study presents an ML-based model to predict maize hybrid yields using breeding genotype characteristics and meteorological variables from trial locations. The model’s output will help in the targeted evaluation of cultivars, allowing for better decision-making in breeding programs.

The primary goal of this study is to develop a predictive model for maize hybrid yields in specific regions using machine learning techniques. This study focuses on three main objectives: (1) to estimate the breeding values of different maize hybrids using the Best Linear Unbiased Prediction (BLUP) method, which is crucial for predicting hybrid yield; (2) to compare the performance of different machine learning models in predicting maize yields, identifying the most reliable approach; and (3) to support practical decision-making by identifying the best regions for planting specific genotypes and forecasting cultivar yields across various regions. By integrating both breeding and environmental data, this research bridges an important gap in crop yield prediction and contributes to the development of more effective breeding strategies.

## 2 Materials and methods

This study aims to predict maize yield using machine learning techniques by incorporating various environmental and breeding factors. The approach follows these steps:

1. Data Collection: The experiment relies on the input breeding value of each hybrid and environmental data, which include variables such as T2M (temperature), TMAX (maximum temperature), TMIN (minimum temperature), PRECTOT (total precipitation), WS2M (wind speed at 2 meters), RH2M (relative humidity), and others. These predictors are linked to maize yield outcomes and serve as the foundational data for model training.
2. Machine Learning Models: Four machine learning models were trained and used for prediction: Random Forest (RF), Extreme Gradient Boosting (XGBoost), Support Vector Regression (SVR), and Gaussian Process Regression (GPR). These models were selected for their ability to handle complex, non-linear relationships between environmental inputs and maize yield. The models were trained on historical data to learn the patterns of yield prediction.
3. Model Interpretability: To enhance interpretability, the relative importance of the predictors was analyzed using the trained models. This step helps in understanding which environmental factors have the greatest influence on the predicted maize yield. The importance of each predictor is visualized in a bar chart, where variables like temperature and precipitation stand out as key influences.
4. Model Evaluation: The performance of each model was evaluated using scatter plots that compare the predicted yields against the actual yields. These plots help visualize how well each model fits the data, with a focus on reducing errors in prediction.
5. Future Yield Prediction: The best-performing model was then used to predict future maize yields for the next 30 years (2025–2054). These predictions are visualized as a time series graph, providing a forecast of maize yield trends over the coming decades, taking into account the environmental variables and their predicted changes.

### 2.1 Experimental materials and data sources

**Figure 1:**
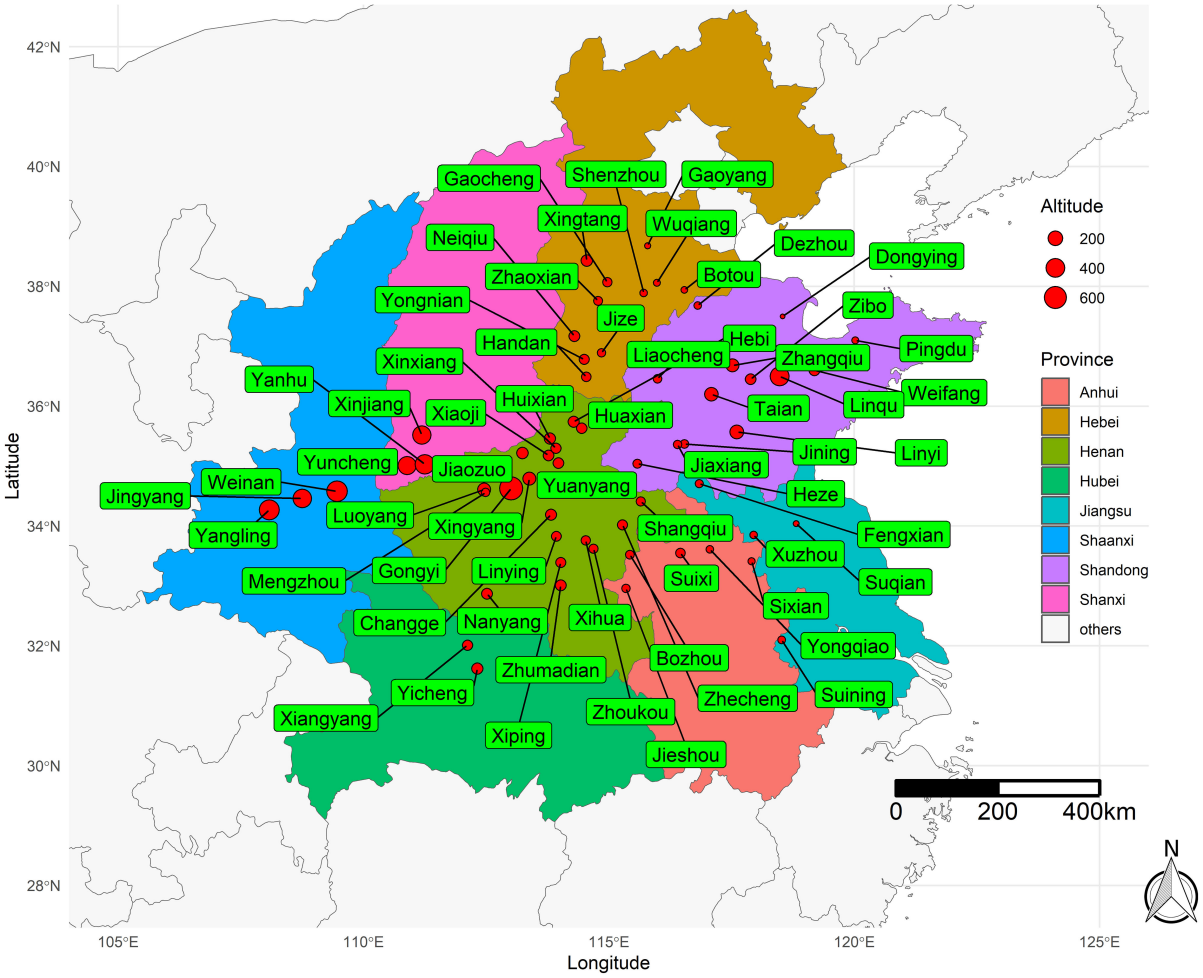
Map of the study area.

This study was conducted in the major maize-producing area of mainland China — the Huang-Huai-Hai (HHH) Plain (**Figure 1**). This region encompasses Shandong, Henan, southern Hebei, southern Shanxi, Guanzhong, and southern Shaanxi, as well as northern Jiangsu and northern Anhui, representing approximately 31.86% of the total national maize cultivation area (Yue et al., 2022).

**Figure 2:**
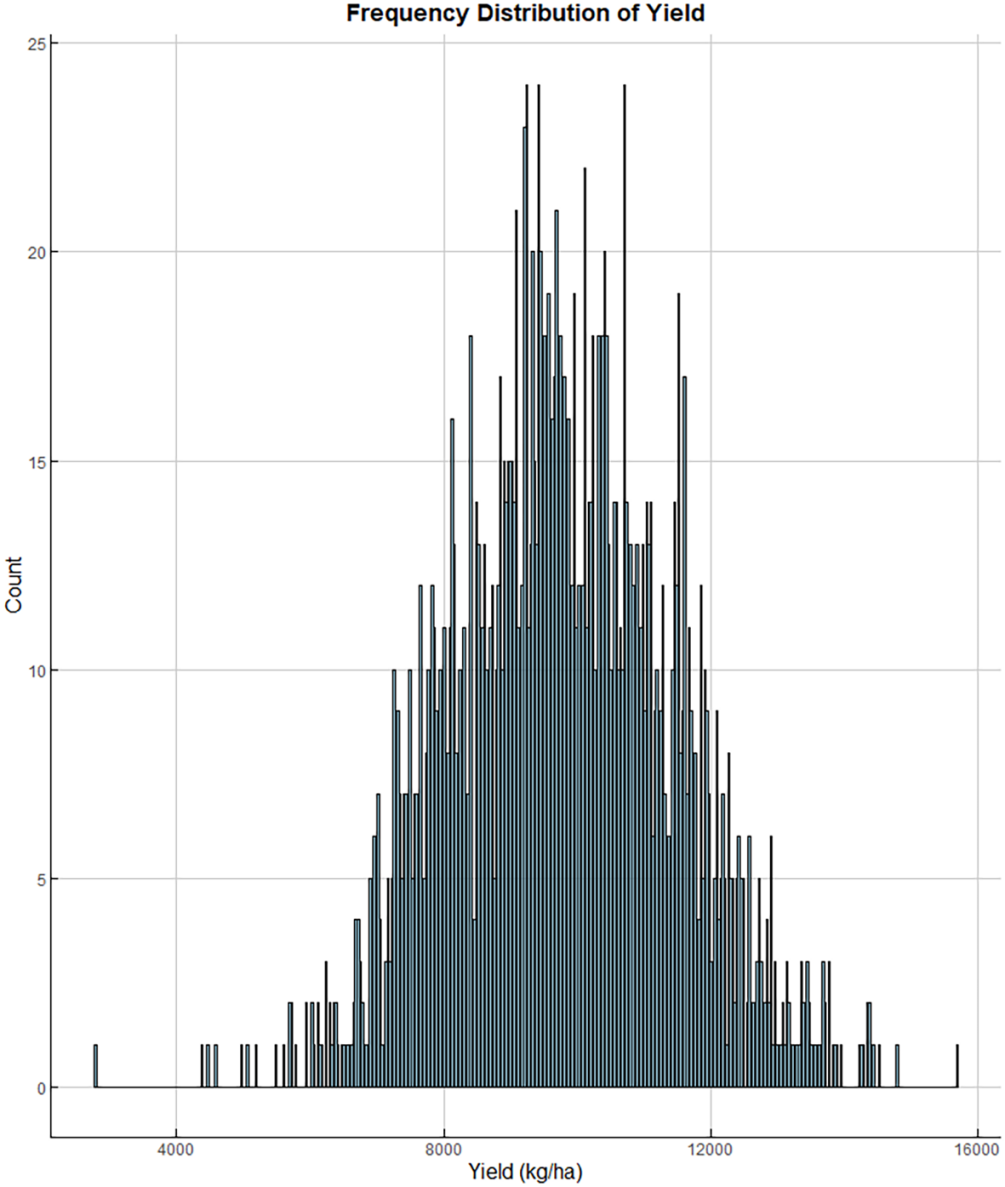
Histogram of the yield frequency distribution from 2016 to 2019.

This study developed a model based on yield data from regional trials of 64 locations and 57 maize hybrids in the HHH ecological region of China, spanning from 2016 to 2019. The histogram illustrating the yield frequency distribution across all trials is presented in **Figure 2**. Field trials were coordinated by the Grain and Oil Crop Research Institute of the Hebei Academy of Agriculture and Forestry Sciences, which supplied both the yield and meteorological data. Each experimental plot consisted of five rows, 6.7 meters long, with a spacing of 0.6 meters between rows. The total plot area was 20.1 square meters (6.7 m × 3 m). Sowing took place from late May to early June, with a planting density of 75,000 plants per hectare, and the experimental design followed a randomized block pattern with three replications. The crop was harvested between late September and early October. Throughout the growing season, agronomic practices were carried out following local field management standards. The maize yield prediction model was developed using meteorological data from the 64 trial locations spread across various counties. This meteorological dataset, which included 19 types of time-series variables (**Table 1**), was obtained from weather observation stations in these counties. The average meteorological values during the growth period were calculated on the basis of the growth cycle of each hybrid and used as input variables for the model.

**Table 1:** The environmental factors used in this research.

Environmental factor	Unit
All sky insolation incident on a horizontal surface ASKSW	MJ m−2 d−1
Downward thermal infrared (longwave) radiative flux ASKLW	MJ m−2 d−1
Extraterrestrial radiation RTA	MJ m−2 d−1
Wind speed at 2 m above the surface of the earth WS2M	m s−1
Minimum air temperature at 2 above the surface of the earth TMIN	°C d −1
Average air temperature at 2 above the surface of the earth T2M	°C d −1
Maximum air temperature at 2 above the surface of the earth TMAX	°C d −1
Dew-point temperature at 2 m above the surface of the earth T2MDEW	°C d −1
Relative air humidity at 2 above the surface of the earth RH2M	%
Rainfall precipitation PRECTOT	mm d −1
Temperature range TRANGE	°C d−1
Potential evapotranspiration ETP	mm d−1
Deficit by precipitation PETP	mm d−1
Vapor pressure deficit VPD	kPa d−1
Slope of saturation vapor pressure curve SPV	kPa °C d−1
Effect of temperature on radiation-use efficiency FRUE	from 0 to 1
Growing degree day GDD	°C d−1
Actual duration of sunshine n	h
Daylight hours N	h

### 2.2 Definition and calculation of breeding value

The additive effect represents the genetic impact that can be reliably transmitted to progeny, as described by quantitative genetics theory. This effect is quantified through the breeding value, which measures the cumulative impact of the alleles an individual possesses (Müller et al., 2018). The breeding value reflects the overall average contributions of all genes within an individual's genetic makeup (Wang et al., 2019). Generally, genotype × environment (G × E) interactions are characterized by the combined effects of genotype (G), location (L), and trial year (tY), as well as the interactions between genotype and location (G × L), genotype and year (G × tY), year and location (tY × L), and genotype, location, and year (G × L × tY). These interactions are often examined using a mixed linear model framework (Yue et al., 2025), as shown in Equation 1.

(1) $$Y=\mu +\text{t}Y_{k}+L_{j}+G_{i}+tYL_{kj}+tYG_{ki}+LG_{ji}+tYLG_{kji}+e$$

where Y denotes the observation of a specific hybrid during trial year k at location j; μ represents the overall mean; $tY_{k}$​ indicates the effect associated with the *k*th trial year; $L_{j}$ signifies the effect of the *j*th trial location; $G_{i}\;$ reflects the effect of the *i*th hybrid, which corresponds to the breeding value of that genotype; $tYL_{kj}\;$ captures the interaction effect between trial year and location; $\;tYG_{ki}$ denotes the interaction effect between trial year and genotype; $LG_{ji}$​ represents the interaction effect between trial location and genotype; $tYLG_{kji}$ indicates the interaction effect among trial year, location, and genotype; and *e* is the random error term. In the model, $L_{j}$ and $tY_{k}$ are fixed effects and random effects, respectively (Fan et al., 2007).

**Figure 3:**
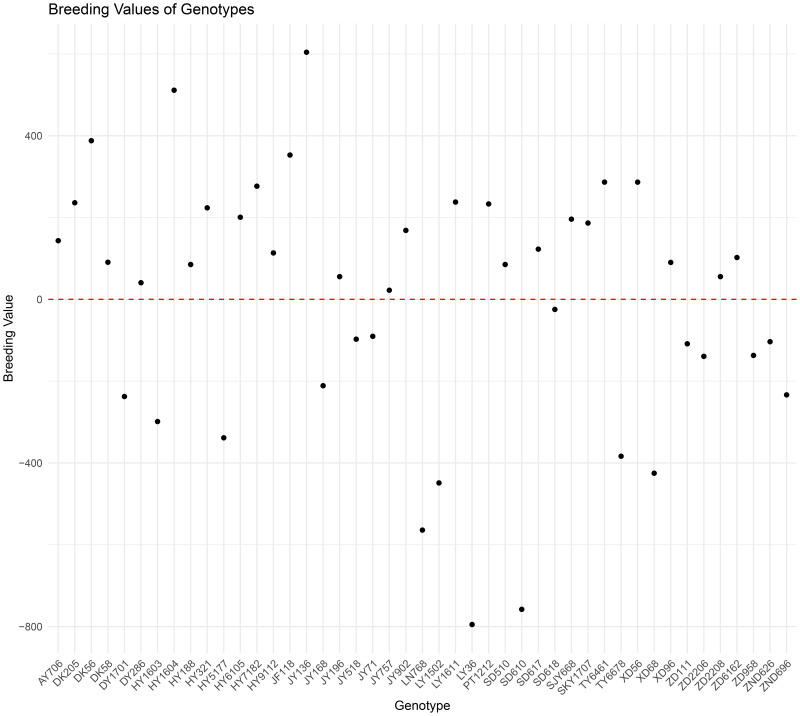
The breeding values of the tested genotypes in the HHH area.

The best linear unbiased prediction (BLUP) is a widely recognized method for estimating the random effects within a mixed linear model. Originally developed for animal breeding to evaluate breeding values, this technique has since been extensively applied in plant breeding and genotype evaluation (Piepho et al., 2008). In this study, the breeding values obtained for various maize hybrids were used to construct a model for forecasting maize yields. Using multilocation trial data collected from maize hybrids in the HHH region between 2016 and 2019, the breeding value for each hybrid was computed using the lmer function from the lme4 package in R software (Bates et al., 2015). The breeding values for each maize hybrid were shown in the form of a scatter plot (**Figure 3**).

### 2.3 Modelling methodology

#### 2.3.1 Random forest model

The RF algorithm (Breiman, 2001) was used to model nonlinear relationships between maize yield and meteorological features. The number of trees (n_estimators = 100–1000), maximum depth (max_depth = 3–20), and minimum samples per leaf (min_samples_leaf = 1–5) were optimized *via* grid search combined with 10-fold cross-validation. Furthermore, RF provides reliable estimates of test errors with minimal computational overhead during model training. A notable advantage of the RF model is its ability to assess feature importance, which identifies the most influential variables in the dataset. In this study, feature importance was evaluated using the “%IncMSE” metric, which measures the change in model accuracy (quantified by the mean squared error) when the values of a given variable are randomly shuffled (Benos et al., 2021). Numerous studies have demonstrated that RF generally outperforms other machine learning techniques in agricultural research.

#### 2.3.2 Extreme gradient boosting

XGBoost is a powerful machine learning algorithm based on gradient boosting that sequentially builds models by adding new trees to correct the errors of the existing ensemble (Niedbała, 2019). Each new tree is trained to predict the residual errors of the combined output from all previously constructed trees. The final prediction is obtained by summing the outputs of all trees in the model. To prevent overfitting and enhance performance, XGBoost incorporates a regularization term. The algorithm minimizes a specified loss function, such as the mean squared error (MSE) for regression tasks, through gradient descent optimization. Given its high predictive accuracy, scalability, and computational efficiency, XGBoost is particularly well-suited for large-scale applications, such as maize yield prediction.

#### 2.3.3 Support vector regression

Support Vector Regression (SVR) is a regression extension of the Support Vector Machine (SVM) designed for predicting continuous outcomes (Speiser et al., 2019). SVR aims to approximate the data within a specified margin of tolerance, known as the epsilon (∈\epsilon∈) margin. The model minimizes the error while ensuring that most data points are within this margin. To make predictions, SVR maps the input features into a higher-dimensional space using a kernel function, such as the radial basis function (RBF) kernel, which enables the model to capture complex, nonlinear relationships between the input features and the target variable. The regularization parameter CCC controls the trade-off between maximizing the margin and minimizing the error. SVR is particularly effective for small to medium-sized datasets and excels in modelling nonlinear patterns in the data.

#### 2.3.4 Gaussian process regression

Gaussian Process Regression (GPR) is a probabilistic model for regression tasks that provides both predictions and uncertainty estimates. GPR models the target variable as a realization of a multivariate Gaussian distribution, where each observed data point is correlated with others on the basis of a covariance function (kernel). This function determines the smoothness and scale of the relationships between the input features and the target variable. GPR computes the posterior distribution over the function space, allowing the model to provide not only a prediction but also the uncertainty associated with that prediction. The model's flexibility in capturing complex nonlinear relationships makes it well-suited for maize yield prediction, especially when the data are noisy or when the underlying function is highly uncertain. The main challenge of GPR is its computational complexity, as it requires the inversion of a covariance matrix, which can become expensive for large datasets (Zhang et al., 2020).

#### 2.3.5 Metrics used to assess the models

In the process of model evaluation, the coefficient of determination (*R*^2^), root mean square error (RMSE), the relative root-mean-square error (RRMSE), mean absolute error (MAE), and mean absolute percentage error (MAPE) were adopted as evaluation metrics. *R*^2^ is a statistical measure that represents the proportion of the variance in the dependent variable that is predictable from the independent variables. It is computed as the ratio of the explained variance to the total variance. This metric is defined in Equation 2. *R*^2^ ranges from 0 to 1, where 1 indicates that the model perfectly explains the variance of the dependent variable, and 0 indicates that the model explains none of the variance. The RMSE is the square root of the mean of the squared differences between the observed values and the predicted values. It measures the average magnitude of the prediction error and is defined in Equation 3. The RMSE reflects how much error there is in the predictions, with lower RMSE values indicating better predictive accuracy. RRMSE is the RMSE normalized by the range or mean of the observed values, allowing for easier comparison across different datasets or models and is defined in Equation 4. RRMSE provides a relative measure of prediction error, making it easier to assess the performance of models with different scales. MAE is the average of the absolute differences between the observed values and the predicted values. It provides a straightforward measure of how far off predictions are, without considering the direction of the errors. This metric is defined in Equation 5. The MAE gives a simple and interpretable value that reflects the average magnitude of the errors, with smaller values indicating better model performance. The MAPE measures the mean absolute percentage errors between the observed values and the predicted values. It expresses the prediction error as a percentage, making it easier to understand the relative error, and it is defined in Equation 6. The MAPE provides a percentage-based error measure, with lower values indicating better model accuracy.

(2) $$R^{2}=1-\frac{\sum_{i}{\left(y_{i}-{\hat{y}}_{i}\right)}^{2}}{\sum_{i}{\left(y_{i}-\bar{y}\right)}^{2}}\;$$

(3) $$RMSE=\sqrt{\frac{1}{n}\sum_{i}{\left(y_{i}-{\hat{y}}_{i}\right)}^{2}}$$

(4) $$RRMSE=\frac{RMSE}{mean(y)}$$

(5) $$MAE=\frac{1}{n}\sum_{i}|y_{i}-{\hat{y}}_{i}|$$

(6) $$MAPE=\frac{1}{n}\sum_{i}|\frac{y_{i}-{\hat{y}}_{i}}{y_{i}}|\times 100\%$$

where n, $y_{i}$, and ${\hat{y}}_{i}$ represent the *i*th number of observations, *i*th predicted value, and *i*th mean of the observed values, respectively.

Each machine learning model underwent hyperparameter tuning using grid search and cross-validation. For the RF model, parameters such as number of trees (n_estimators), maximum depth (max depth), and minimum samples per leaf (min_samples_leaf) were optimized.

### 2.4 Experimental environment and statistical software

The computer information used in this research is as follows: operating system: Microsoft Windows 10 Home (Version 10.0.19045); processor (CPU): Intel Core i5-1035G1 @ 1.00 GHz (with a boost to 1190 MHz); BIOS: Insydel F.06, 2020/4/8; system manufacturer: HP; System Model: HP Pavilion Laptop 15-cs3xxx; and RAM: 8.00 GB. All the statistical analyses used in this study were conducted using R version 4.3.1, with breeding values using the lme4 (v1.1-34) package and model predictions for RF, XGBoost, SVR, and GPR in machine learning using the xgboost (v1.7.5.1), e1071 (v1.7-13), and kernlab (v0.9-32) packages, respectively.

## 3 Results

### 3.1 Evaluation of the four machine learning models

The dataset was randomly divided into training (80%) and test (20%) subsets, containing 1,676 and 420 samples, respectively. Each model underwent hyperparameter optimization using the training data, and the optimized model was subsequently validated on the test dataset. Model performance was evaluated using the statistical metrics *R*², RMSE, RRMSE, MAE, and MAPE (**Table 2**). All four models—Random Forest (RF), Extreme Gradient Boosting (XGBoost), Support Vector Regression (SVR), and Gaussian Process Regression (GPR)—demonstrated acceptable predictive capability for maize yield, with *R*² values exceeding 0.5. The RF model achieved the best overall performance. It obtained an *R*² of 0.79 and an RMSE of 812.4 kg ha^-^¹ on the training dataset, and corresponding values of 0.64 and 1010.6 kg ha^-^¹ on the independent test dataset. The *R*² value of 0.79 for the RF model was calculated based on the comparison between the predicted and observed yields within the training dataset itself, reflecting the in-sample fit of the model. The moderate decline in performance (approximately 24% increase in RMSE) between the training and test datasets is within the expected range for machine learning models, indicating that the RF model maintained strong generalization capability without overfitting. According to Niedbała et al. (2019), models with MAPE < 9% can be classified as highly accurate. The RF model achieved *R*² = 0.64, RMSE = 1010.59 kg ha^-^¹, RRMSE = 10.32%, MAE = 743.89 kg ha^-^¹, and MAPE = 8.25%, qualifying it as highly accurate. Although XGBoost, SVR, and GPR performed slightly worse, all models achieved R² > 0.5, confirming their predictive utility.

**Table 2:** Exponential comparison metrics of the four forecasting models used in this study.

Models	Metrics				
	*R* ^2^	RMSE (kg/ha)	RRMSE (%)	MAE (kg/ha)	MAPE (%)
RF	0.64	1010.59	10.32	743.89	8.25
XGBoost	0.54	1141.99	11.66	841.54	9.22
SVR	0.53	1152.34	11.77	863.14	9.73
GPR	0.52	1157.43	11.82	892.61	9.94

*R*^2^, coefficient of determination; RMSE, root mean square error; RRMSE, relative root mean square error; MAE, mean absolute error; MAPE, mean the absolute percentage error; FR, random forest; XGBoost, extreme gradient boosting; SVR, support vector regression; GPR, Gaussian process regression.

**Figure 4:**
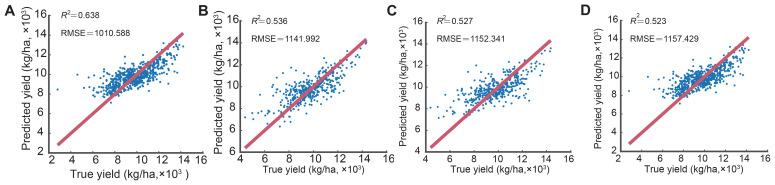
Comparison between observed and predicted maize yields for the four machine learning models: **(A)** RF, **(B)** XGBoost, **(C)** SVR, and **(D)** GPR.

**Figure 4** presents scatterplots comparing the observed and predicted maize yields for the four models. The red line represents the 1:1 reference line, where perfect predictions would lie. The color of each point indicates the trial year (2016–2019), and the point size reflects the number of observations per experimental location. To maintain visual clarity, 95% confidence intervals are not shown, but model uncertainty was quantitatively evaluated using the statistical indicators in **Table 2**. The RF model exhibited the closest alignment of points to the 1:1 line and the smallest residual dispersion, confirming its superior predictive reliability and stability across different environments.

### 3.2 Relative importance of predictors in estimating maize yields

**Figure 5:**
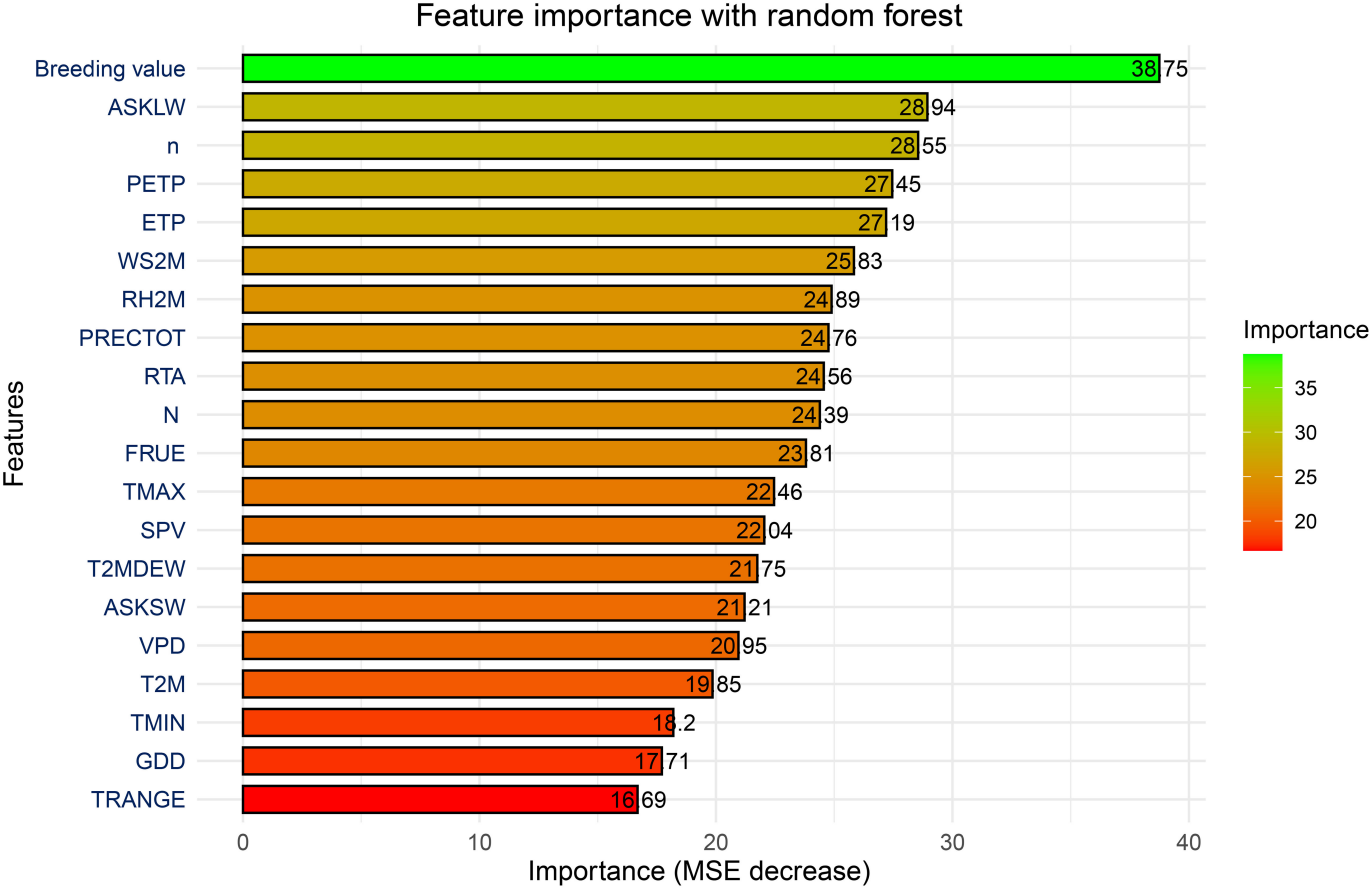
The relative importance of predictors in estimating yield based on the RF model.

To explore which environmental factors have the greatest impact on the model's regression prediction ability, we used MSE decrease to visualize the importance of these environmental factors in this study. As shown in **Figure 5**, key predictors influencing maize yields within the RF model can be identified through variable importance analysis. These predictors are assessed using the "percentage increase in the mean square error" (MSE%) metric, where a greater increase in MSE% signifies a more influential variable. The findings revealed that breeding value stands out as the most critical factor affecting maize yields. This breeding value reflects the combined effect of genes, which significantly impacts yield determination. Among the meteorological variables, downwards longwave radiation flux (ASKLW), sunshine duration (n), precipitation deficit (PETP), and potential evapotranspiration (ETP) were identified as the most significant contributors to maize yields, followed by and surface-level wind speed (WS2M). In contrast, the temperature range (TRANGE) had the smallest effect on yield predictions among all the variables.

**Figure 6:**
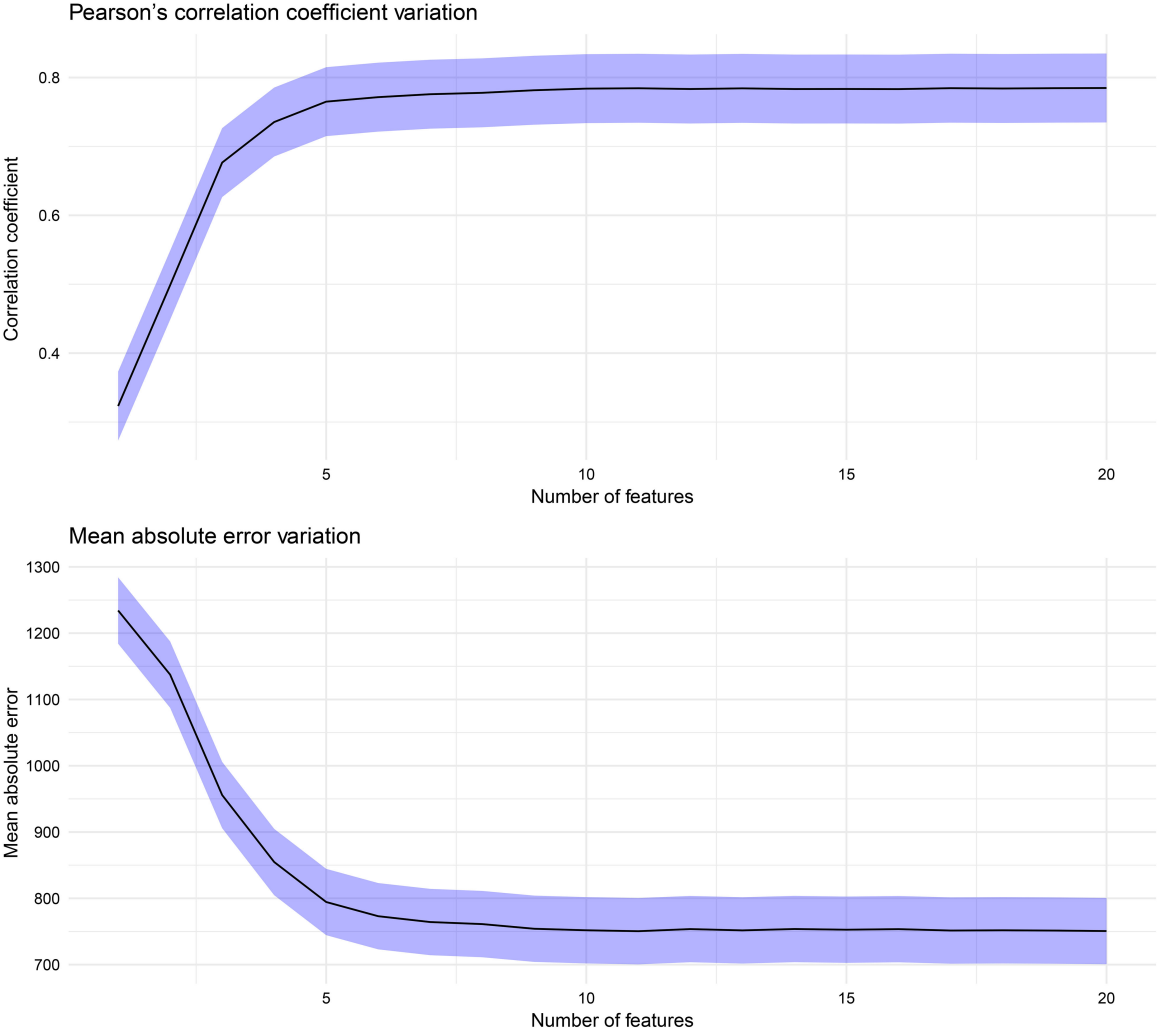
Variation in the Pearson’s correlation coefficient and mean absolute error (MAE).

Before delving into the specifics of the performance of our machine learning models, it is important to understand the variations in the predictive accuracy as the number of features in the dataset increases. **Figure 6** shows the variations in the correlation coefficient (r) and mean absolute error (MAE) as the feature set size changes, one feature at a time, according to the ranking presented in **Figure 4**. The increases in r and decreases in the MAE are not uniform; instead, a notable peak occurs with the top five features: the breeding value, ASKLW, n, PETP, and ETP. When the random forest (RF) model utilizes these five features for forecasting maize yield, it achieves an explanatory power of over 75% (with an MAE of approximately 800 kg ha^−1^). In contrast, the model’s predictive performance decreases to approximately 50% (with an MAE exceeding 1000 kg ha^−1^) if the features n, PETP, and ETP are excluded.

### 3.3 Partial dependence profiles (PDPs) for the random forest model

**Figure 7:**
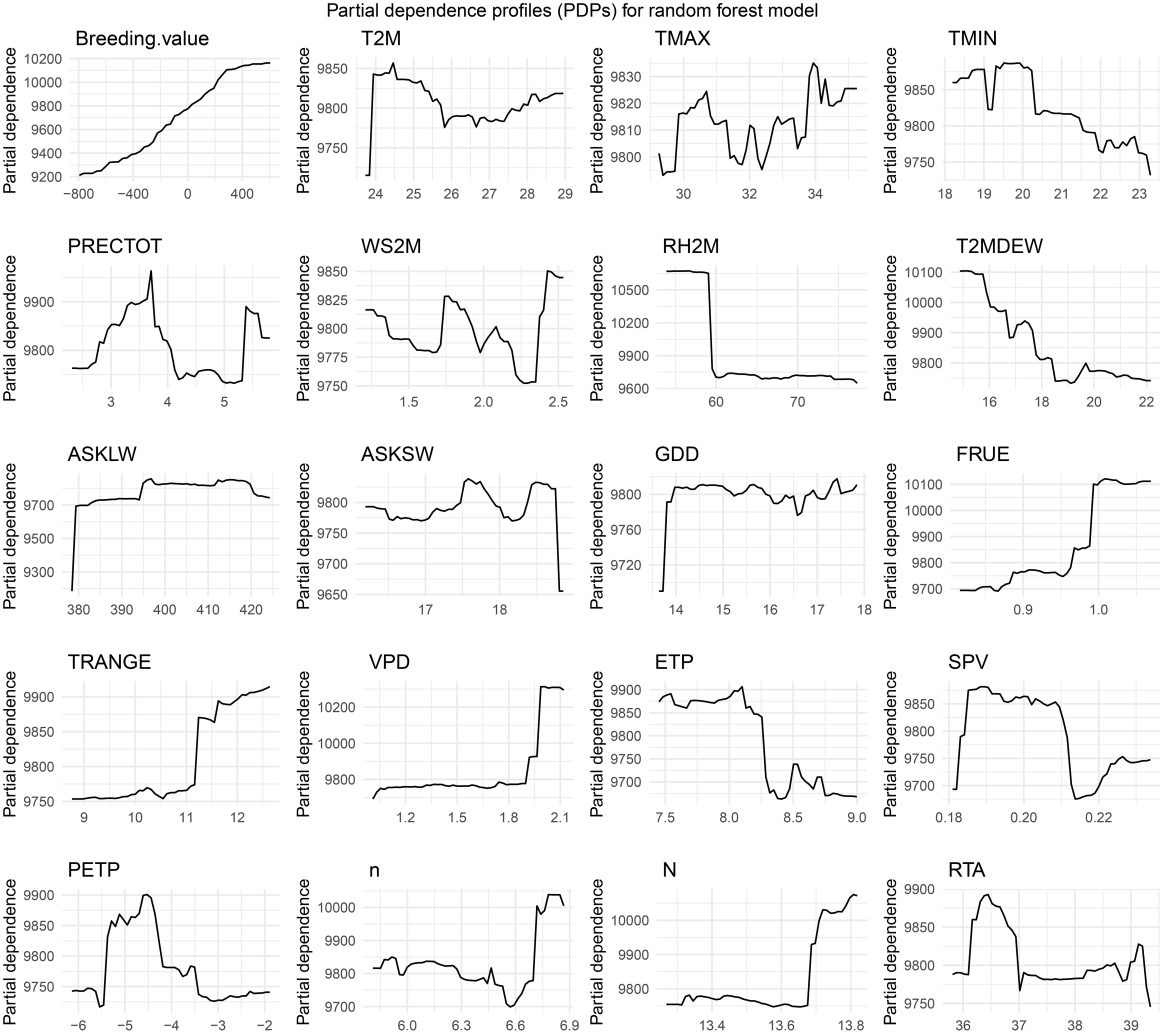
Partial dependence profiles (PDPs) for 20 features of the RF model. Please refer to **Table 1** for the meaning of abbreviations for the meteorological factors.

We explore the partial dependence profiles (PDPs) of various features included in our random forest model. These plots provide valuable insights into how each feature influences the model's predictions, with other variables held constant. By analysing the shape and trends of these curves, we can identify the most influential variables and their relationships with the predicted yield. This analysis helps to highlight key features that contribute significantly to the model’s performance, and guide further feature engineering or refinement of the model (**Figure 7**). According to the PDP results, an increase in breeding value leads to a higher predicted yield for maize hybrids. However, when the daily average temperature (T2M) exceeds 24.5°C d^−1^, the maximum temperature (TMAX) exceeds 31°C d^-1^, the minimum temperature (TMIN) exceeds 19°C d^-1^, and the dew point temperature (T2MDEW) exceeds 16°C, the yield decreases. A relative humidity (RH) greater than 60%, downwards thermal infrared (longwave) radiative flux (ASKLW) greater than 380 MJ MJ m^−2^ d^−1^, all-sky insolation incident on a horizontal surface (ASKSW) below 19 MJ m^−2^ d^−1^, and growing degree days (GDD) exceeding 14°C d^−1^ have minimal impacts on yield. Conversely, rainfall precipitation (PRECTOT) above 5 mm and a wind speed at 2 m above the surface of the earth (WS2M) above 2.25 m/s result in yield reduction. As a short-day plant, maize has little variation in yield when the daily photoperiod is shorter than 13.6 hours. Extraterrestrial radiation (RTA) above 37 MJ m^−2^ d^−1^ also has a negligible effect on yield variation. In contrast, vapor pressure deficit (VPD) below 2.0 kPa d^−1^ and saturation vapor pressure (SPV) below 0.19 kPa d^−1^ both contribute to an increase in yield.

### 3.4 Maize production forecast between 2025 and 2054

**Figure 8:**
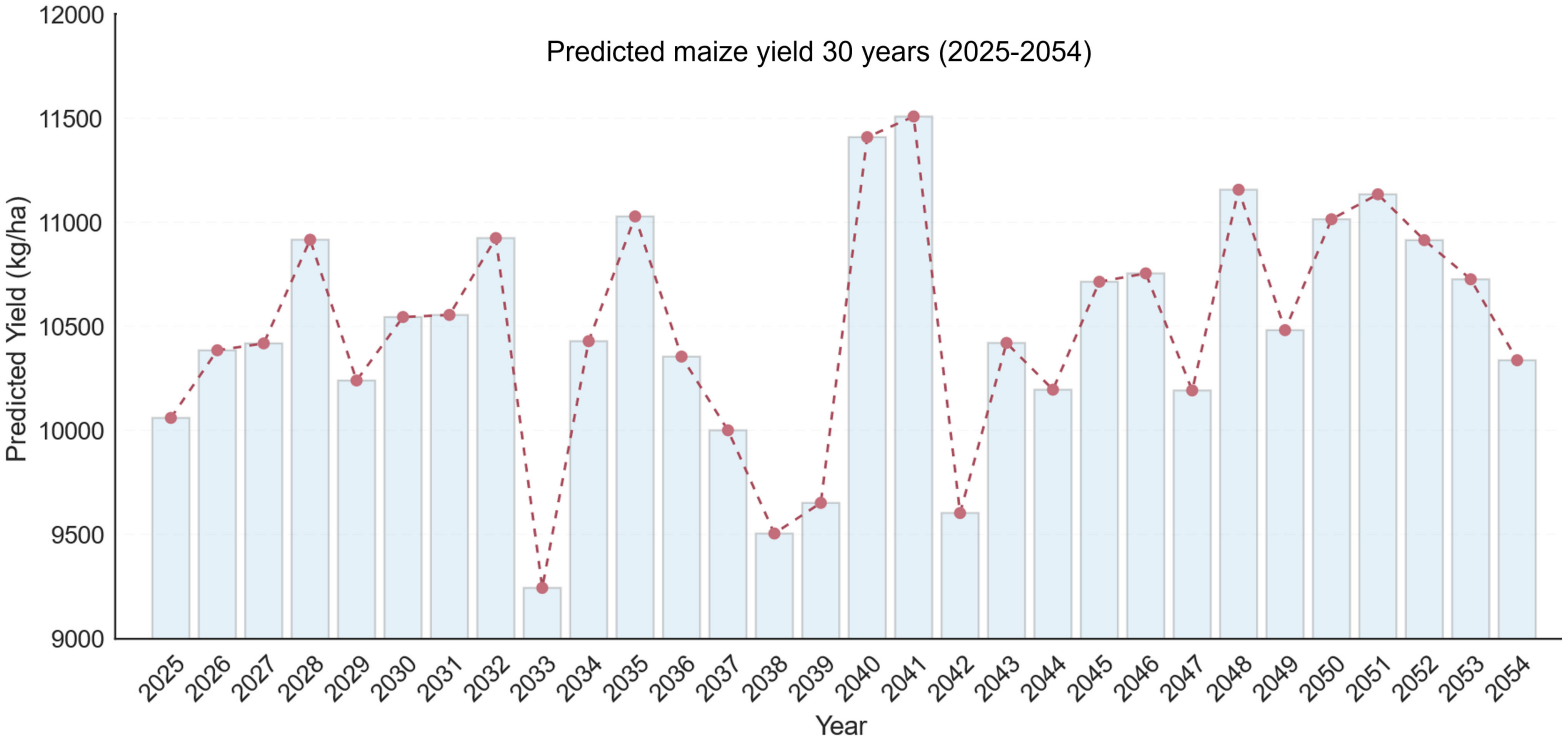
Predicted maize yield based on random forest from 2025 to 2054.

Using the optimized RF model, maize yields from 2025 to 2054 were projected based on meteorological and genetic inputs derived from the 2016–2019 dataset (**Figure 8**). The forecast indicates noticeable interannual variation throughout the 30-year period. The highest predicted yield (11,508 kg ha^-^¹) occurs in 2041, whereas the lowest yield (9,245 kg ha^-^¹) is expected in 2033. The production decline in 2032–2033 corresponds to years with severe precipitation deficit (PETP > 90 mm) and maximum temperatures above 32 °C, implying intensified drought and heat stress during grain filling. In contrast, the yield increase in 2041–2042 aligns with moderate solar radiation (ASKSW ≈ 21 MJ m^-^² day^-^¹) and optimal growing degree days (GDD ≈ 12–14 °C day^-^¹), which promote photosynthesis and kernel development. These findings suggest that fluctuations in future maize yield are primarily driven by temperature extremes and moisture imbalance, emphasizing the need for adaptive breeding and management strategies under changing climatic conditions.

## 4 Discussion

### 4.1 Comparative performance of machine learning models

We prioritized algorithms with a strong track record on tabular agro-environmental data—Random Forest (RF), XGBoost, Support Vector Regression (SVR), and Gaussian Process Regression (GPR)—because they balance accuracy, robustness, and interpretability in breeding and management contexts. Across the held-out test set in our study, RF delivered the highest accuracy (*R*² = 0.64; RMSE = 1010.6 kg ha^-^¹; MAPE = 8.25%), outperforming XGBoost, SVR, and GPR. To place this in context, our RF performance aligns well with independent maize-yield studies that report mid-range explanatory power (*R*²≈0.50–0.70). For example, using city-level statistics and multi-source environmental data across China, Chen et al. (2021) reported RF correlation R ≈ 0.806 for maize, corresponding to R² ≈ 0.65—essentially the same explanatory level as our test-set RF (*R*² = 0.64). Likewise, a county-scale satellite–meteorology EC-LUE framework on the North China Plain reproduced 64% of the variance in maize yield at county scale (R² = 0.64), and province-stratified analyses in the same study showed *R*² = 0.57 (Henan) to 0.58 (Shandong), i.e., within the same performance band as our model (Hai et al., 2023). Together, these references indicate that achieving *R*² around 0.60 with region-scale, multi-environment datasets is both realistic and competitive for operational yield prediction. By identifying high-yield hybrids tailored to specific environmental conditions, farmers can optimize their cultivation strategies, increasing productivity and sustainability in maize production (Cairns et al., 2012; Erenstein et al., 2022). Additionally, the insights gained from the variable importance analysis can guide breeders in prioritizing the most influential traits, such as breeding value and specific meteorological parameters, further refining the development of high-yielding hybrids (Xu et al., 2020; Farooq et al., 2024).

Why did RF outperform XGBoost, SVR, and GPR here? First, RF can capture heterogeneous, nonlinear interactions between breeding value and weather covariates without heavy feature engineering, while remaining relatively insensitive to hyperparameter misspecification. Second, RF’s built-in out-of-bag/aggregation reduces variance and mitigates overfitting on moderate-size, noisy multi-location trial data. This is consistent with prior agronomic findings that tree-based ensembles are reliable baselines for regional maize yield forecasting under mixed genetic–environmental drivers (Jeong et al., 2016). From a practical standpoint, our RF result (*R*² = 0.64) compares favorably with these peer studies in the same R² band (≈0.57–0.65) while additionally providing feature importance that breeders can act on (e.g., breeding value and key hydro-radiative variables). In sum, within the commonly observed 0.50–0.70 R² range for regional maize yield prediction, our model sits near the upper end and offers interpretable diagnostics useful for cultivar recommendation and management planning.

### 4.2 Importance of predictors in maize yield estimation

The variable importance analysis revealed that breeding value is the most critical predictor of maize yield. This finding aligns with the fundamental role of genetic traits in determining crop performance (Crossa et al., 2010). Among the meteorological factors, downwards longwave radiation flux (ASKLW), sunshine duration (n), precipitation deficit (PETP), and potential evapotranspiration (ETP) emerged as the most significant contributors to yield prediction. These findings corroborate previous studies emphasizing the importance of radiation and water availability in crop development (Jones et al., 2003; Lobell et al., 2014). Interestingly, temperature-related variables, such as the temperature range (TRANGE), were found to have minimal influence on yield predictions. This contrasts with previous studies (Tao and Zhang, 2013) that identified temperature as a major determinant of maize growth. The minimal impact observed in this study may be due to the relatively narrow range of temperature variability in the data or the genetic adaptation of the hybrids used in the trials. Further investigations are needed to assess the interactions between temperature and other environmental variables in maize production.

The partial dependence plots (PDPs) provided valuable insights into the relationships between the predictors and maize yield. The results highlight the nonlinear nature of these relationships, with thresholds for key variables such as temperature, precipitation, and wind speed. For example, yield reductions were observed when the maximum temperature (TMAX) exceeded 31°C, emphasizing the detrimental effects of heat stress on maize productivity. Similar patterns were observed for excessive precipitation and wind speed, which is consistent with the findings of Liu and Basso (2020). On the other hand, some predictors, such as relative humidity (RH) greater than 60% and extraterrestrial radiation (RTA) above 37 MJ/m²/d, had negligible effects on yield. These results suggest that not all environmental variables have a linear or significant effect on maize production. This highlights the importance of feature selection and model interpretability in machine learning applications for agriculture.

### 4.3 Future yield trends and implications

The RF model’s projections for 2025–2054 revealed pronounced year-to-year fluctuations in maize yield, reflecting climate variability and its influence on crop growth. A yield reduction of approximately 15.4% occurred between 2032 (10,924 kg ha^-^¹) and 2033 (9,245 kg ha^-^¹), coinciding with increased precipitation deficit (PETP > 90 mm) and elevated maximum temperatures (> 32 °C)—conditions indicative of combined drought and heat stress. Similarly, a 16.5% decline from 2041 (11,508 kg ha^-^¹) to 2042 (9,604 kg ha^-^¹) corresponds to similar climatic extremes. Conversely, yield surges of 18–19% between 2033–2035 and 2039–2040 were linked to more favorable radiation and temperature conditions, particularly ASKSW ≈ 21 MJ m^-^² d^-^¹ and GDD between 12–14 °C d^-^¹.

These findings are consistent with previous global climate change assessments (Rosenzweig et al., 2014), confirming that maize productivity will likely experience both opportunities and challenges under future climatic variability. Importantly, the developed modeling framework—integrating genetic (breeding value) and climatic data—provides a practical tool for regional yield forecasting and adaptive management. The approach could be extended to other major maize-producing regions such as the U.S. Corn Belt, South America, and sub-Saharan Africa, or even to other cereal crops (e.g., wheat, rice, sorghum) that share similar physiological responses to environmental stressors. Such transferability would facilitate broader applications of data-driven decision support in global food systems.

### 4.4 Limitations of the study and future research directions

With the rise of foundation models and pretrained architectures in computer vision and natural language processing, it is indeed possible to adapt such models for agricultural prediction tasks. However, most existing foundation models are designed for unstructured data and have not been tailored to structured, tabular formats such as breeding datasets. While transfer learning holds promise, its application to BLUP and genotype-environment datasets remains limited and requires careful domain-specific adaptation. Future work will explore the integration of pretrained models or hybrid frameworks, particularly when multi-modal data (e.g., imagery, environmental sensors) are available. Such extensions may further enhance prediction performance, especially in data-scarce regions.

While this study demonstrates the efficacy of machine learning approaches in yield prediction, it is essential to acknowledge certain limitations. The model's performance depends inherently depends on the quality and comprehensiveness of the input data. Future research should aim to incorporate a broader range of environmental variables, including soil health metrics to enhance the model's predictive capabilities. Additionally, integrating real-time data sources such as satellite imagery and IoT sensors could facilitate more dynamic yield predictions that account for immediate changes in environmental conditions (Sumathi et al., 2022). Additionally, the study focused solely on maize hybrids; thus, extending the research to other crop species could provide a more comprehensive understanding of the applicability of machine learning models in diverse agricultural systems. Exploring the interactions between various crops and environmental factors may yield valuable insights into crop rotation strategies, further contributing to improved food security.

## 5 Conclusion

This study developed a machine learning-based framework for predicting maize yields, with the random forest (RF) model (with an *R*^2^ value of 0.64) demonstrating superior performance compared with Extreme Gradient Boosting (XGBoost), Gaussian Process Regression (GPR), and Support Vector Regression (SVR). The analysis highlighted the importance of breeding value, and key meteorological variables were highlighted, providing actionable insights for breeders and farmers. Partial dependence plots revealed nonlinear relationships between the predictors and yield, underscoring the complexity of maize production systems. The predictive capabilities of the model were further validated through future yield forecasting, highlighting the potential impact of climatic variability on maize production. By facilitating the selection of high-yield hybrids suited to specific environments, the proposed approach offers a cost-effective and efficient solution for intelligent breeding and precision agriculture. Future research should focus on expanding the dataset to include more diverse environmental conditions and hybrid types and should explore the integration of remote sensing data to increase model performance.

## Data Availability

The raw data supporting the conclusions of this article will be made available by the authors, without undue reservation.
